# Smart Patch for Skin Temperature: Preliminary Study to Evaluate Psychometrics and Feasibility

**DOI:** 10.3390/s21051855

**Published:** 2021-03-06

**Authors:** Heejung Kim, Sunkook Kim, Mingoo Lee, Yumie Rhee, Sungho Lee, Yi-Rang Jeong, Sunju Kang, Muhammad Naqi, Soyun Hong

**Affiliations:** 1 College of Nursing, Yonsei University, Seoul 03722, Korea; hkim80@yuhs.ac; 2 Mo-Im Kim Nursing Research Institute, Yonsei University, Seoul 03722, Korea; 3 Multifunctional Nano Bio Electronics Lab, Department of Advanced Materials and Science Engineering, Sungkyunkwan University, Suwon 16419, Korea; seonkuk@skku.edu (S.K.); kangsj@skku.edu (S.K.); alinaqi@skku.edu (M.N.); 4 Korea Electronics Technology Institute, Seongnam 13509, Korea; emingoo@keti.re.kr (M.L.); slee@keti.re.kr (S.L.); 5 Department of Internal Medicine, Endocrine Research Institute, College of Medicine, Yonsei University, Seoul 03722, Korea; YUMIE@yuhs.ac; 6 Department of Nursing, Samsung Medical Center, Seoul 06351, Korea; yirang.jeong@samsung.com

**Keywords:** remote sensing technology, body temperature, feasibility study

## Abstract

There is a need for continuous, non-invasive monitoring of biological data to assess health and wellbeing. Currently, many types of smart patches have been developed to continuously monitor body temperature, but few trials have been completed to evaluate psychometrics and feasibility for human subjects in real-life scenarios. The aim of this feasibility study was to evaluate the reliability, validity and usability of a smart patch measuring body temperature in healthy adults. The smart patch consisted of a fully integrated wearable wireless sensor with a multichannel temperature sensor, signal processing integrated circuit, wireless communication feature and a flexible battery. Thirty-five healthy adults were recruited for this test, carried out by wearing the patches on their upper chests for 24 h and checking their body temperature six times a day using infrared forehead thermometers as a gold standard for testing validity. Descriptive statistics, one-sampled and independent *t*-tests, Pearson’s correlation coefficients and Bland-Altman plot were examined for body temperatures between two measures. In addition, multiple linear regression, receiver operating characteristic (ROC) and qualitative content analysis were conducted. Among the 35 participants, 29 of them wore the patch for over 19 h (dropout rate: 17.14%). Mean body temperature measured by infrared forehead thermometers and smart patch ranged between 32.53 and 38.2 °C per person and were moderately correlated (r = 0.23–0.43) overall. Based on a Bland-Altman plot, approximately 94% of the measurements were located within one standard deviation (upper limit = 4.52, lower limit = −5.82). Most outliers were identified on the first measurement and were located below the lower limit. It is appropriate to use 37.5 °C in infrared forehead temperature as a cutoff to define febrile conditions. Users’ position while checking and ambient temperature and humidity are not affected to the smart patch body temperature. Overall, the participants showed high usability and satisfaction on the survey. Few participants reported discomfort due to limited daily activity, itchy skin or detaching concerns. In conclusion, epidermal electronic sensor technologies provide a promising method for continuously monitoring individuals’ body temperatures, even in real-life situations. Our study findings show the potential for smart patches to monitoring non-febrile condition in the community.

## 1. Introduction

Body temperature is a physical measurement of the basic functions of the human body. Body temperature has been widely used to detect or monitor health conditions [1]. In general, the normal range for body temperature for a healthy adult is between 36.5 and 37.2 °C (97.8–99 °F). An individual’s body temperature can differ by about 0.5 °C (0.9 °F) between its highest and lowest points every day [1]. The human body has multiple thermoregulatory mechanisms, when exposed to extreme heat and cold, the physiological capacity operates a mechanism to keep the temperature within the normal range [2]. Abnormal body temperature means below or above normal ranges of temperature. Body temperature drops below normal in cold environments where the body loses heat faster than it can produce. The temperature below normal range can lead to impaired mental function or specific failures in circulation [1]. Symptoms of below normal range temperature include memory loss, shivering, decreased levels of consciousness, or confusion [3]. As the hypothalamus controls body temperature, it can reset at a higher body temperature its response to an infection or other febrile conditions [1]. The temperature above a normal range can lead to dehydration, fatigue, headache, and confusion, and it can be life-threatening [1]. Thus, it is very important to maintain body temperature within an optimal range.

Body temperature can be measured in a variety of instruments. Traditionally, body temperature has been measured using contact thermometers, which are placed on the oral, axillar, esophagus or rectum [4]. Every thermometer has unique pros and cons. The oral temperature is one of the commonly use thermometers among contact measurements. The oral temperature is measured when the silver tip on the end of the thermometer is under the tongue and the lips wrapped around the instrument [1]. The reading may be abnormal if a person has recently smoked, eaten cold, hot drinks or food [1]. Axillary thermometry is very easy to measure compared to oral or rectum thermometers [5,6,7]. However, the effect of the environmental conditions and incorrect placement of the thermometer indicates low sensitivity and specificity to fever detection [5]. Accurate body core temperature can be measured in the esophagus or rectum [8,9] with specific limitations. In particular, long-term use of these thermometers is financially costly and calibration checks may be required to improve measurement accuracy [10]. Thus, it is inconvenient to use widely in real life. While non-contact infrared thermometers are an instrument that has been frequently used recently due to pandemic monitoring [11]. This thermometer can be measured rapidly without any contact [11,12]. Non-contact infrared thermometers do not cause person-to-person infection, has cost-effectiveness and require no preparation prior to measurement [11,12]. Regardless of indoor exercise and recovery, forehead temperature has a smaller bias with wide limits of agreement when compared to oral, anal and axillary temperatures, because the temporary arteries are connected to the jugular arteries [6,11,12]. Thus, non-contact infrared thermometers are more efficient than contact thermometers in real life situation.

During the COVID-19 pandemic, it is recommended that people use a non-contact method of measuring body temperature through thermal imaging systems and non-contact infrared thermometers in public healthcare settings [13]. Non-contact infrared thermometers can reduce cross-contamination risks or the spreading of infection between people [13,14]. In general, it is easy and quick to use to apply non-contact temperature measurement to multiple places on the body in both adults and children. However, some scientific evidence has raised some questions about the effectiveness of the non-contact method of measuring body temperature. First, it can be difficult to confirm clinical validation to detect infections, especially those that do not exhibit obvious hyperthermia [15]. Second, non-contact infrared thermometers are easily influenced by external factors in the indoor and outdoor environment [16]. That is infrared thermographic imaging is highly affected by time of day, ambient temperature and relative humidity. Third, incorrect measurements frequently occur due to the distance and angle between thermal camera and object [17] because public healthcare practitioners do not follow manufacturers’ instructions for use, including installation, operation and training [13].

To overcome the limitations of both contact and non-contact based measurements, patch-type body thermometers have been developed with the demand by public healthcare practitioners and researchers [18,19]. Patch-type body thermometers are able to provide continuously monitoring body temperature as a clinical and assistive tool to check for immediate temperature changes [18,19]. When the patch correctly positions and firmly attach the body parts, patch-type body thermometers can be easy to measure an individual temperature pattern using accessory programs, for example of mobile applications (apps) [18,19]. Thus, patch-type body temperature has been recognized as a useful alternative or supplement to collect objective, continuous and real-time patient data instead of direct care for healthcare informatics [18,19]. In addition, these temperatures emphasize the features of continuous data collection with sensors that can detect multiple types of data and communicate this data with ubiquitous data storage [18,19]. Thus, the end-users, either healthcare providers or consumers, have better access to the data and have the benefit of monitoring repeatedly measured data for the monitoring and predicting targeted conditions [18,19].

However, there have been few attempts to evaluate the usability and feasibility of such thermometers in practical situations. The usability of patch-type body thermometers should be evaluated to determine both the validity of their ability to detect specific body sensor data and the feasibility of their real-world application. However, several prior studies evaluating body temperature sensors have been mainly focused on the device’s function, i.e., the devices validity [20,21]. Most previous studies were conducted in engineering disciplines, thus, they strongly focused on how they developed the device and tested the accurate collection of data using new sensors and advanced technology [20,21]. Moreover, they conducted their experiments in a laboratory setting, which is very controlled and free from the extraneous variables. Although some studies tested human subjects, they included very homogenous subjects within the same occupational groups [22,23] and clinical patients with similar health conditions [24,25,26]. Thus their study finding has evitable limitations of the generalizability.

To facilitate the clinical application and user adaptation of the device, it is necessary to evaluate usability considering the human–device interaction and potential barriers that may influence the consumer experience in daily life [27]. Additionally, the physiological sensing of the human body is having the utmost importance in the healthcare field, which requires a smart sensing system that can synchronously respond and collect data of the physical signal of the human body with a high integration system to obtain accurate and precise long-term monitoring. Considering this, we introduced a wearable sensor system to measure the body temperature in real-time with high accuracy and error-free measurements. Thus, this study used smart patches developed by this multidisciplinary research team. A previous study has confirmed the highly sensitive and responsive performance of the smart patch [28], however, there is a gap between the laboratory environments and the real world for body temperature. This multidisciplinary study aimed to: (1) describe the process to develop the smart patches to measure the body temperature of adults living in their real environments; (2) evaluate the reliability and validity by comparing the body temperature measured by the infrared forehead thermometer and (3) report usability including user experience of smart patches in daily life.

## 2. Materials and Methods

### 2.1. Study Design

This study was cross-sectional and descriptive in design, using one-group time-series design.

### 2.2. Ethical Consideration

The Institutional Review Board of an affiliated university approved the study (Approval no. Y-2017-0031). In addition, all participants were provided with information on the voluntary nature of the study, their freedom to withdraw their enrollment, and the strategies that would be adopted to protect their anonymity and confidentiality, after which written informed consent was collected.

### 2.3. Study Participants

A convenience sample of 35 subjects was recruited when they determined that their health status was good to participate in daily activities and this study on the basis of their declaration. Inclusion criteria are to: (1) be between 19 and 64 years old; (2) have use of a smartphone or tablet computer; (3) be currently living without significant health problems requiring medication; (4) have the ability to sustain the smart patch on the upper chest without a known skin allergy and (5) have the ability to read and understand Korean. Exclusion criteria are (1) patients in the hospital; (2) pregnant and lactating women; (3) those participating in other research projects and (4) those with serious depression or cognitive impairment because of inaccurate operation or poor compliance [24]. All participants who assessed eligibility met the inclusion criteria. This study aimed to assess feasibility rather than statistical significance; no formal sample size calculation was performed. However, compared to the previous study proposed sample size of 30 was considered adequate to meet the objectives of the study and recommend a sample size of 12–30 individuals for feasibility studies [24,29].

### 2.4. Measures

**Figure 1:**
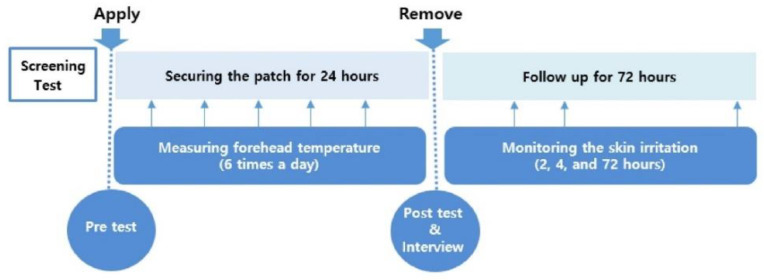
Study procedures.

When initiating the experiment, participants completed self-reporting questionnaires, which asked questions including age, sex, living conditions, occupation and environment. The study participants were requested to check their body temperature with an infrared forehead thermometer, specifically the Dotori Multi FS-201 (HuBDIC, Anyang, Korea), to confirm the concurrent validity of the smart patch as a gold standard in this study. Forehead temperature measured with this infrared thermometer is known to be as valid as axillary temperature measured with mercury-in-glass thermometers [30,31,32]. Study participants checked their forehead temperature, they were asked to record the time, place, ambient temperature and humidity, the presence of sweat and daily activity on a paper-based checklist at an interval of every 3-h. After completing the instructed measures for 24 h, the participants were interviewed and answered two 5-point Likert-type questions (1 = strongly disagree, 5 = strongly agree) by asking about the comfort and usefulness of the device. Research assistants with the nursing license checked participants’ skin condition immediately after removing the smart patch. After 1, 2, 4 and 72 h from removal, follow-up observations were conducted by taking picture of the sites to check for any skin irritation or adverse events due to the device. All experimental procedures are illustrated in Figure 1.

#### Description of Smart Patch for Skin Temperature

**Figure 2:**
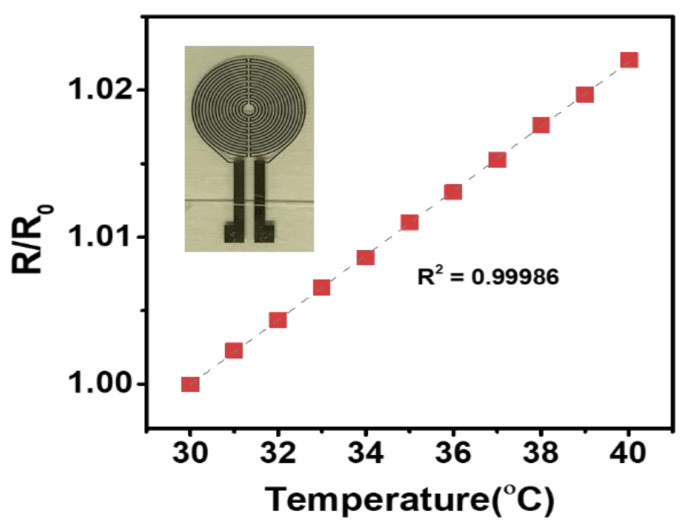
Sensor response (30–40 °C with an interval of 1 °C).

**Figure 3:**
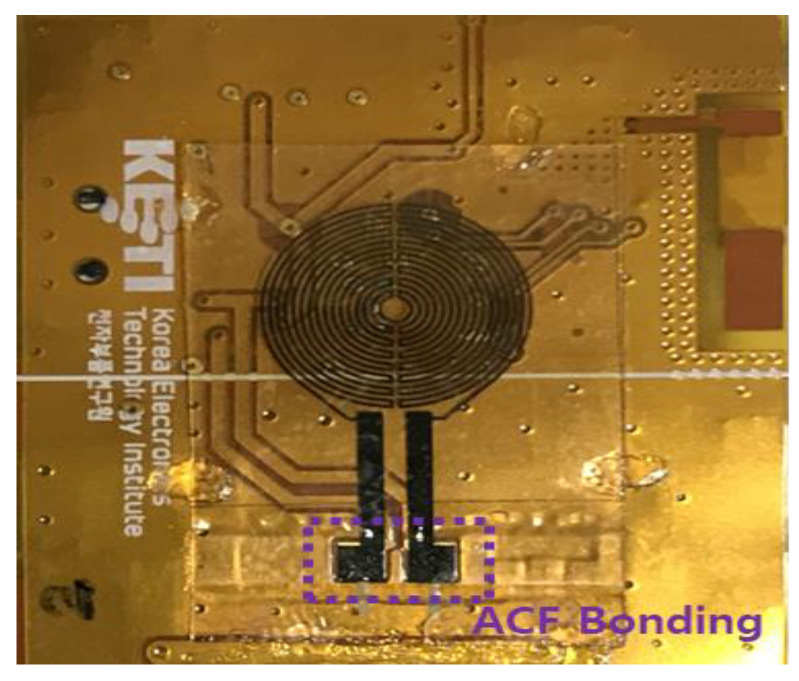
Sensor and circuit bonding (anisotropic conductive film (ACF) bonding).

**Figure 4:**
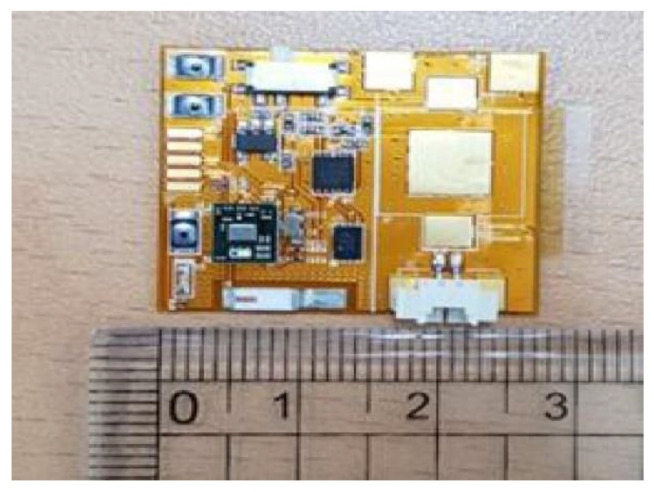
Sensor circuit (enlarged view)**.**

**Figure 5:**
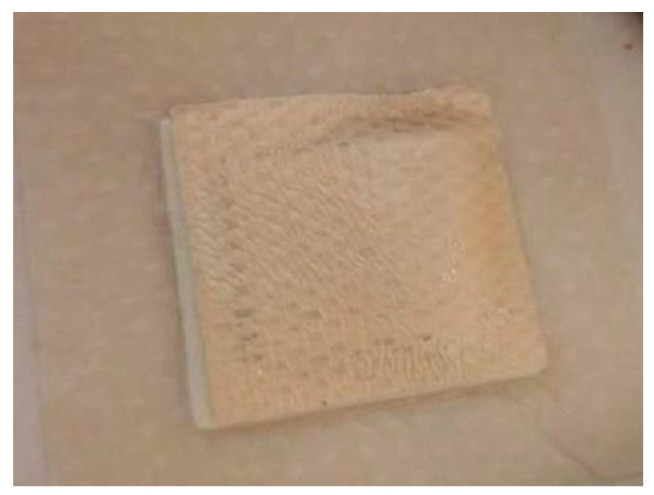
Securing the smart patch.

The skin temperature-measuring smart patch used for this study was developed in the Multifunctional Nano Bio Electronics laboratory of Professor Sunkook Kim at the affiliated university and manufactured as a prototype by T&L Co., Ltd. and Korea Electronics Technology Institute. As the engineering details were reported [28], the platinum (Pt) as a sensing material is utilized and patterned by a simple lithography method [28], shown in the inset of Figure 2. The resistance response of the proposed temperature sensor was measured under different temperature levels ranges from 30 to 40 °C, revealing stable linear characteristics as shown in Figure 2. Additionally, to establish accurate and error-free monitoring of body temperature, the anisotropic conductive film (ACF) bonding technique was utilized for bonding the sensor part to the circuit part (see Figure 2 and Figure 3) [28]. This device not only detects microbiological signals, such as body temperature, from a person’s body surface but also is also flexible and compact (40 mm × 40 mm). It is composed of four parts: (1) a multichannel platinum-resistance thermometer; (2) a signal-processing integrated circuit; (3) a wireless communication module and (4) an elastic battery. Functionally, this attaching-type, wearable device was set on a 1-min sensing cycle, thus battery capacity continued for approximately 72 h per single charge. Attached on the skin, the smart patch collects and transmits data regarding body temperature to Google Cloud and via Bluetooth to an individual’s smart phone concurrently. Based on this information sharing system, data were able to be stored automatically and monitored by researchers continuously at distance (see Figure 4 and Figure 5).

### 2.5. Data Collection

Participants were recruited through word-of-mouth and flyers posted on bulletin boards at companies, public health centers, campuses and community centers located in Seoul, Korea. Study participants were enrolled after providing informed consent. The general study protocol was designed based on the previous study [24]. The smart patch was applied to the left side of the upper chest similar to the previous study [24]. Although this smart patch was able to attach to other body parts, such as axilla, thigh or back, the upper chest has shown to cause less discomfort in sweaty conditions, during sleep and during activities and is reported to show similar levels of forehead temperature [33]. The smart patch was attached laterally to the sternum at the height of the first or intercostal space of the participant’s chest. After attaching the patch, a film dressing, which is generally used for wound care, was applied over the smart patch for effective attachment and protection against moisture. The trained research assistants gave instructions for proper securement to each participant. The data were collected using standardized self-reporting questionnaires, observation notes and unstructured, 5-min interviews conducted from September 2018 to February 2019. The participants received compensation equivalent to USD $5 for their time and effort.

### 2.6. Data Analysis

Descriptive statistics were used to illustrate the data at each observation point. Paired *t*-tests or independent *t*-tests were used to compare temperatures measured by two thermometers in terms of differences in temperature according to a user’s position, ambient temperature and humidity. The Kolmogorov–Smirnov test was performed to confirm the normality of data prior to the correlation analysis. Since all data series demonstrated normal distribution, the Pearson’s correlation coefficients were used to confirm similarities between two different body temperatures. One-sampled and independent *t*-tests and Bland-Altman plots were used for examine the difference between two body temperatures and subsequently observe the measurement errors and their confidence intervals. Multiple linear regression was conducted with infrared forehead body temperature as an independent variable, and ambient temperature and humidity as covariates. In addition, checking receiver operator characteristic curve was used for evaluating area under the curve explained, sensitivity, specificity and positive and negative predictive values regarding two different cutoffs. Finally, the responses of participating subjects were quantitatively summarized with mean (standardized deviation, SD) and qualitatively described through content analysis.

## 3. Results

### 3.1. Description of the Study Participants

The average age of the 35 participants was 30.29 (SD = 9.63) years; they were mostly female (20/35, 57.1%), had a bachelor’s or higher degree (23/35, 65.7%) and most were college students (9/35, 25.7%) or graduate students (4/35, 11.4%). Six participants (dropout rate = 17.14%) were excluded due to incomplete data secondary to: (1) device errors of data storage or (2) partially missing information in spite of 24-h attachment of the smart patch. The sample characteristics of those excluded from the analyses did not significantly differ from those of the 29 participants included in the final analyses.

### 3.2. Comparison between Infrared Forehead and Smart Patch Body Temperatures

**Figure 6:**
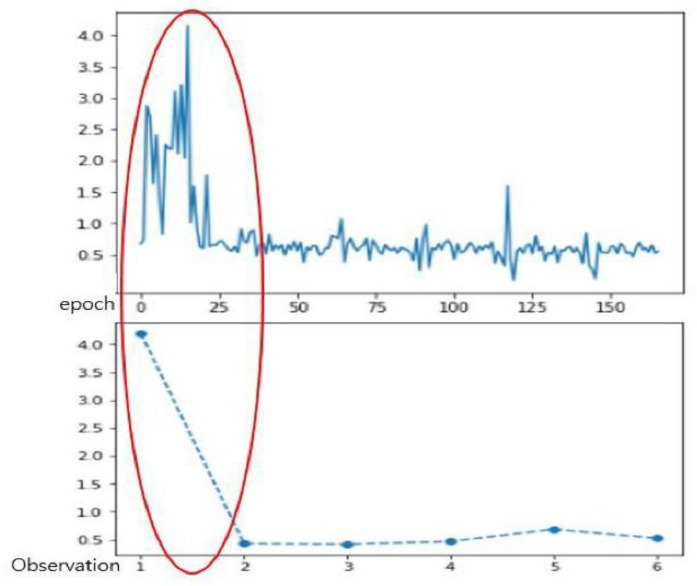
Temperature variability over the measurement.

Individual traces of infrared forehead and smart patch body temperatures throughout the day are provided in Appendix A. As a referent, the average of infrared forehead temperatures ranged from 36.58 to 38.2 °C (SD = 0.29–0.43). However, the body temperatures measured by the smart patch ranged from 32.53 to 36.98 °C (SD = 0.70–4.60). Based on the 165 total measurements across 29 participants, there were significantly lower temperatures measured by the smart patch than the infrared forehead temperatures (M_diff_ = −0.61, SD = 2.56, t = −3.04, *p* = 0.003) with a small correlation (r = 0.12, *p* = 0.128). In the one-sampled *t*-test, there was statistical difference between two body temperatures among the matched observations in the pooled sample (*p* < 0.001). The average body temperature measured by the smart patch (M = 36.074, SD = 0.75) was higher than that measured by the infrared forehead temperatures (M = 36.61, SD = 0.77). When comparing each observation time, the largest mean differences between two measures were at Times 1 and 6, and the lowest correlation between two body temperature were at Time 5 (Table 1 and Figure 6).

### 3.3. Consistency between Two Body Temperatures

**Figure 7:**
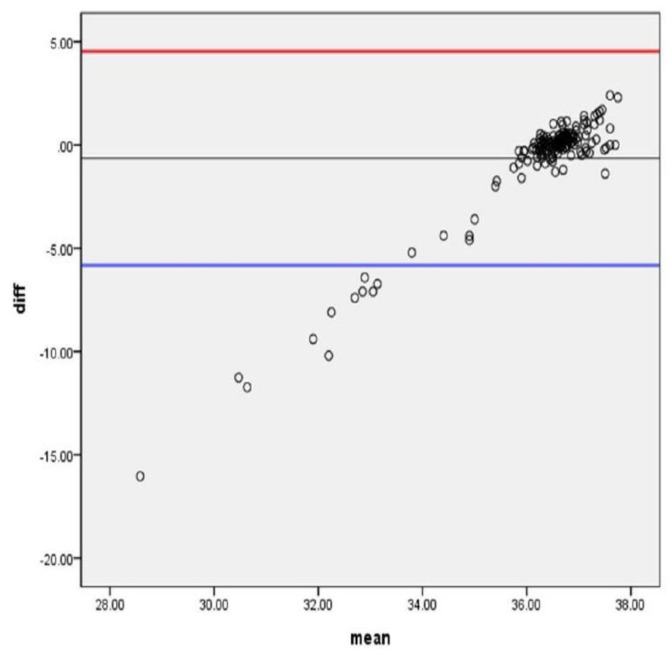
The Bland-Altman plot of mean differences.

Figure 7 shows the Bland-Altman plot [34], which showed the distribution of mean differences between the two thermometers. Bland-Altman plots showed strong agreement between the two temperatures of measurement that 94% of the mean difference was located within the acceptable limits of one standard deviation (95% upper limit = 4.52; lower limit = −5.82). Approximately, 6.6% were found out of the acceptable agreement range because they were below the lower limit.

### 3.4. Considerations of Users’ Intrapersonal Characteristics

The level of participants’ physical activity was self-reported. The majority of respondents were in the sitting position (60.61%), standing position (15.9%) and supine position (10.6%) when checking the body temperature. Other physical activities included walking (3.3%), rest (2.4%), eating (2.4%), work (2.4%), shower (0.5%), drinking (0.5%), cleaning (0.5%), running (0.5%) and stretching (0.5%). Most users (*n* = 106, 74.6%) reported a sitting position when measuring the body temperatures based on two different thermometers. The mean difference (SD) of each component of position was as follows: sitting position −0.72 (±2.70), supine position −0.19 (±3.06) and standing position −0.53 (±2.60). However, there were no significant mean differences between two body temperatures by each position (Table 2).

### 3.5. Smart Patch Body Temperature Explained by Users’ Environmental Characteristics

The linear regression model was built with smart patch body temperature as a dependent variable, infrared forehead body temperature as an independent variable, and ambient temperature and humidity as covariates. Using the data at all 133 observations across Times 1–6 after listwise deletion, R-squared was 0.046 with 0.742 square root of the sum of the error at squared. Further regression was tested after deleting observations at Times 1, 5 and 6 because the results of *t*-test, correlation efficient and Bland-Altman plot showed some systemic measurement errors existing. In the model with the data at Times 2–4, R-squared was 0.125 with 0.581 square root of the sum of the error at squared based on the data at 83 observations at Times 2, 3 and 4. Both ambient temperature and humidity were not significantly associated with smart patch body temperature (Table 3).

### 3.6. Diagnostic Validity to Detect the Febrile Condition

We compared two cutoffs to decide the febrile conditions based on 37.5 °C cutoff of the World Health Organization’ quarantine guide (2020) [35]. Table 4 shows the sensitivity, specificity, positive predictive value and negative predictive value among 136 valid data points in the pooled observations. When the febrile condition was set at 37.3 °C, the specificity and negative predictive value were 0.84 and 0.96 respectively, however the sensitivity and positive predictive value were very low. After this, when the febrile condition was set at 37.5 °C, the specificity and negative predictive value improved, while the sensitivity and positive predictive value were still problematic (Table 4).

### 3.7. User Evaluation

Most of the study participants reported positive responses about the comfort to wear (M = 3.37, SD = 0.81) and ease to maintain (M = 3.49, SD = 0.74). They mentioned satisfaction of “being self-monitored” that the study participants were able to monitor their own body temperature using the supplied mobile app. The study participants perceived how their daily activities and surrounding environment affect the body temperature changes. Thus, they stated that they had some insight to maintain the normal body temperature for the health and safety.

Few participants reported some discomfort due to limited daily activity (*n* = 2), itchy skin (*n* = 7) or detaching concerns (*n* = 2). In some situations, especially when exercise was prolonged, high-intensity and in a hot environment, extra sweat led to increased showering. We provided protective film to the participant to prevent frequent showers from damaging the smart patch and falling off the participant’s body. Participants attached the protective film to the smart patch during the shower and removed it after the shower. However, smart patches also fell off in the process of removing protective films, and, frequent hot water showers have damaged the appearance of the smart patches. Other problems include itching of the attachment site due to skin dryness and discomfort caused by the smart patch when moving during sleep (see Appendix B).

## 4. Discussion

This study aimed to assess the reliability and validity of the smart patch and examine user evaluations to assure the feasibility of using the smart patch in a natural setting. The present findings have confirmed that 94% of the mean difference between the two measures were located within acceptable limits. A smaller variability of data was observed with the smart patch compared to the infrared forehead thermometer. Using 37.5 °C in infrared forehead temperature as a cutoff to define febrile conditions is appropriate to detect that an individual is without significant fever compared to an individual with a high fever. Users’ position while checking and ambient temperature and humidity do not affect the smart patch body temperature. The compliance rate was 85% in terms of data completeness after 100% adhering to the study protocol. However, some concerns were reported in terms of disruption to daily life, such as difficulty with exercise, taking showers and attachment issues. 

The present findings have confirmed that 94% of the mean difference between the two measures were located within acceptable limits. Some outliers were found when the smart patch temperature located below 35 °C. These significant low temperatures may result from some methodological errors of the wearable device itself as similar to the previous study findings [24]. Body temperatures detected by the wearable devices seem to be 3–4 °C lower than that of the nurse’s check. Thus, it is required to handle this discrepancy due to outliers or measurement errors. After handling the outliers, the smart patch may be an alternative to the infrared forehead thermometer in a natural setting. Specifically, a smaller variability of data was observed with the smart patch when compared to the infrared forehead thermometer. The larger variability of data collected by the infrared forehead thermometer resulted from measurement errors among different raters, poor adherence to the measurement protocols or very diverse environmental factors [30]. This stability of repeated data has the strength to monitor body temperature via a noninvasive method continuously, considering heat-related illness and occupational situations [36]. Specifically, an individualized device, such as our smart patch, is helpful in preventing cross infection from direct contact with an infected person [30].

Using 37.5 °C as a cutoff to define febrile condition is appropriate to detect the febrile condition, which is similar to previous studies and the current practice [35]. The sensitivity and positive predictive value were relatively low compared to the clinical criterion to replace an infrared forehead thermometer to define fever-related conditions [37]. However, these low diagnostic values are somewhat expected from the previous studies conducted with patient groups located in relatively well-controlled environments, such as hospitals [30]. To detect febrile conditions, further development and validation of the smart patch is required to improve the accuracy of identifying febrile conditions in excess of 38 °C. Instead, our smart patch may be useful to detect non-febrile conditions for mass surveillance rather than febrile conditions at specific clinics. The further study should be replicated using individuals with high fevers based on deep body temperature to develop implication for diagnosis purposes in community settings [30,32].

The main finding of the current study was that changing the body position or posture did not affect body temperatures measured by the smart patch. It means that the users applying the smart patch can make any position and do not have a limitation in daily activities for this perspective. The usual check of the body temperature in the clinic is completed in the standing position, however checking the body temperature with the specific position at observation times has been questioned to evaluate the validity of certain body temperatures [9]. Although our study finding is preliminary with small sample sizes and tests only two methods, this finding has some clinical inference. Changing the position freely means that the method can be applied to diverse patients with different activity levels. For example, some adults are so active that they can stand or walk, while others may be more likely to sit or lay on the bed due to impaired mobility, such as hemiparesis or hemiplegia. Moreover, infants or very old persons are more likely to lie on the bed when they feel sick due to infectious diseases. However, the position does not make a difference to the body temperature between and within two methods.

We investigated the relationship of body temperatures with ambient temperature and humidity. The smart patch measures relatively stable and accurate temperatures over time than those of forehead thermometers. A previous study explained that the controlled laboratory environments are generally less affected by environmental factors such as ambient temperature, humidity, wind and ambient light than by outdoor environments [9]. However, the smart patch is easy to use for monitoring the body temperature in our living environment and daily activities. Although the smart patch is very thin and flexible, it does not seem to be affected by ambient temperature and humidity. This finding can relate to some clinical inference. Outdoor workers face elevated and prolonged heat exposures and the most serious health and safety issue in this regard is heatstroke [22]. Heat stroke can be fatal if it is not immediately treated [22]. Survival depends on whether they identify the symptoms and get medical help as soon as possible because outdoor workers tend not to notice the symptoms associated with heatstroke by themselves [22]. Thus, the further study may focus on outdoor workers who work in the extreme heat to confirm this possible benefit and may suggest occupational monitoring system with the smart patch to prevent heat-related disease on the worksite.

The compliance rate was 83%, in terms of data completeness, after adhering to the study protocol, while some concern in daily life were reported, such as exercise, taking a shower or attachment issues. In our study, two participants reported some discomfort while sleeping. Improving easiness to use and comfort is very important in a clinical setting [30,32]. As a physical aspect, it is needed to be firmly attached to enhance its utilization over time. The smaller and more flexible device could achieve greater comfort for users. In a functional aspect, it would be helpful to have some features, such as an alarm message sent to the smart phone or a blinking lamp on the patch when the smart patch is detached from the skin. The study participants recommend these features because they want to know whether the patch is working properly and attached well.

There are some aspects related to “human factors” in addition to the above mentioned device and functional factors. In this preliminary study of 35 participants, we found that data from 6 participants was not stored in the smart patch due to some human factors. First, the smart patch error may occur as the result of poor staff training at the beginning of the experiment. Improperly securing or monitoring of the smart patch may result from the staff being inconsistently or poorly trained. Each staff has different levels of skill and capacities to operate the smart patch [38,39]. Thus, we retrained the research assistants with the standardized protocol after the preliminary test with few cases. Second, the participants’ physical activity, excessive sweating or frequent showers can cause errors with the smart patch, thus, factors related to the user may be an important source of device error [40,41]. Third, unfamiliarity of the participant may affect different movement or levels of activities to decrease satisfaction, which is emphasized in the previous study [24]. Prevention of human-related errors is very important to enhance the reliability and usability of the device as human error is generally seen to be a major contributor to the validity and safety of smart wellness devices [40,41]. Therefore, researchers should continue to train staff with standardized protocols and a user’s guide [38,39].

### 4.1. Implication for Clinical Practice

Our smart patch has the ability to improve public health and safety in clinical situations. First, our smart patch’s features may be helpful in monitoring patients in recovery after a fever or infectious disease or isolated individuals in clinical situations [42]. Second, our smart patch may be useful when caring for patients who are sensitive to external stressors. In other words, certain patients, such as infants or hyperactively delirious patients, need to receive minimum levels of direct contact care [43]. Using the smart patch may minimize frequent contact with healthcare providers and unnecessary stimuli, such as waking at night for vital sign checks [24]. Third, the device may be essential in protecting healthcare providers caring for those with contagious diseases [42] by allowing non-contact surveillance or monitoring from a distance. With this device, healthcare providers have less chance to contract a pathogen and less burden of wearing protection gear. This adds to protection from contagion for healthcare providers.

### 4.2. Limitations

There are several limitations to this study. First, we did not use the deep body temperature measured in the esophagus or rectum due to the need for a non-invasive approach, and there is some question of the validity of infrared forehead temperature [36]. Thus, the next study should use multiple and more diverse thermometers to measure body temperature compared to measurements made by the smart patch. Second, there were some extraneous variables, such as work and rest in occupational situations, clothing or exposure to radiators or air conditioners [36]. Thus, future studies should include subgroup analyses within specific occupations or environments. In addition, more diverse types of sensor data can be used to estimate deep body temperature, such as heart rates [36]. To generalize the study findings and utilize the smart patch in an ecological setting, future studies should consider the influence of age, body composition, psychological stressors, circadian rhythms and static exertion [36]. Third, some participants complained about the inconvenience of wearing smart patches. The sense of wearing a smart patch has an impact on long-term wearing of sensors. This smart patch is formed from a suitable, thin and flexible film. However, it is necessary to improve film thickness of smart patch that does not affect the precision of skin temperatures. Finally, the study participants measured their temperatures using infrared forehead thermometers by themselves and some concern exists regarding inter-raters. Thus, a standardized protocol should be provided for the study participants when the next experiments are conducted in a natural setting.

## 5. Conclusions

In conclusion, the present study has confirmed the specificity validity, stability over time and acceptable usability of the smart patch in a natural setting with human subjects. Compared to the infrared forehead thermometer, there is an observed level of agreement to measure epidermal temperature to monitor a non-febrile condition. Our study findings show epidermal electronic sensor technologies provide a promising method for continuously monitoring individuals’ body temperature, even in real-life situations. However, further development and validation of the sensor technique is required to improve the accuracy of predicting febrile conditions in the natural setting. 

## Figures and Tables

**Table 1:** Comparison and correlation between smart patch and infrared forehead body temperatures.

	BT_1_ (Smart Patch)	BT_2_ (Infrared Forehead)	Mean Difference of BT_1_-BT_2_		Correlation of BT_1_ and BT_2_
	M (SD)	M (SD)	M (SD)	t (*p*)	r (*p*)
Time 1	36.58 (0.43)	32.53 (4.60)	−4.05 (4.56)	−4.78 (<0.001)	0.14 (0.482)
Time 2	36.62 (0.39)	36.58 (0.52)	−0.04 (0.57)	−0.38 (0.709)	0.23 (0.236)
Time 3	36.61 (0.29)	36.56 (0.61)	−0.05 (0.60)	−0.47 (0.643)	0.27 (0.168)
Time 4	36.62 (0.29)	36.72 (0.70)	0.10 (0.66)	0.80 (0.432)	0.35 (0.074)
Time 5	36.10 (0.34)	36.89 (1.05)	0.28 (1.12)	1.28 (0.210)	−0.04 (0.856)
Time 6	36.61 (0.33)	36.98 (0.74)	0.37 (0.67)	2.85 (0.009)	0.43 (0.030)

**Table 2:** Differences in mean difference of BT_1_-BT_2_ by position.

	Mean Difference of BT_1_-BT_2_		
Position	*n*(%)	M (SD)	F (*p*)
Sitting	106 (74.6)	−0.72 (2.70)	0.229 (0.796)
Supine	12 (8.5)	−0.19 (3.06)	
Standing	24 (16.9)	−0.53 (2.60)	

BT_1_ = smart patch, BT_2_ = infrared forehead.

**Table 3:** Regression models with infrared forehead body temperature and ambient temperature and humidity.

	*N* = 133 at Times 1–6	*N* = 87 at Times 2–4
	B (*p*)	B (*p*)
Ambient temperature	−0.079 (0.373)	−0.114 (0.337)
Ambient humidity	0.048 (0.596)	−0.163 (0.152)
Infrared forehead body temperature	0.188 (0.033)	0.315 (0.006)
R2 (*p*)	0.046 (0.099)	0.125 (0.014)
Square root of the sum of the error at squared	0.742	0.581

**Table 4:** Comparison of sensitivity, specificity, positive predictive value and negative predictive value, and the receiver operating characteristic (ROC) of the smart patch.

Cutoff	37.3 °C		37.5 °C	
	Value	95% CI	Value	95% CI
Sensitivity	0.50	(0.18, 0.82)	0.50	(0.09, 0.91)
Specificity	0.84	(0.82, 0.86)	0.89	(0.88, 0.91)
Positive predictive value	0.17	(0.06, 0.27)	0.13	(0.01, 0.28)
Negative predictive value	0.96	(0.94, 0.99)	0.98	(0.97, 0.99)
ROC	0.71	(0.53, 0.99)	0.90	(0.85, 0.95)

ROC = receiver operating characteristic.

**Figure A1:**
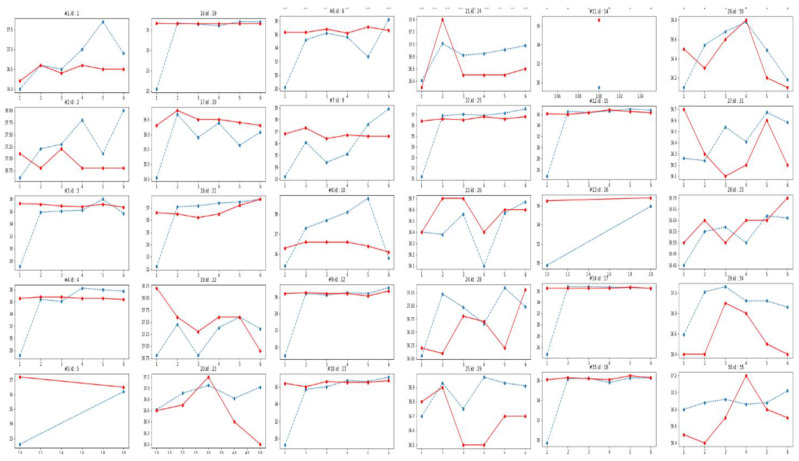
The red line means the body temperature measured by the infrared forehead thermometer; the blue line means the body temperature measured by the smart patch. The fifth graph at the first line is the dropout participant, missing information in spite of the 24-h attachment of the smart patch.

## Data Availability

The data presented in this study are available on request from the corresponding author. The data are not publicly available due to privacy/ethical restrictions.

## Appendix B. Skin Observation before Attaching, during Attacking and after Detaching the Smart Patch

Participant A			
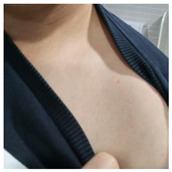
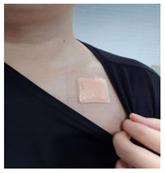
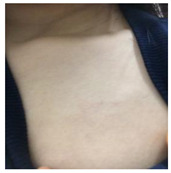
Participant B			
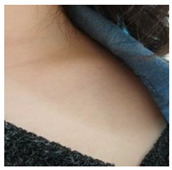
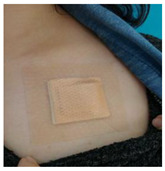
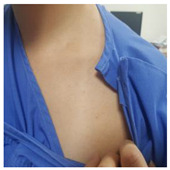
	Before attaching	During attaching	After detaching
